# A Young, Immunocompetent Woman with Small Bowel and Hepatic Mucormycosis Successfully Treated with Aggressive Surgical Debridements and Antifungal Therapy

**DOI:** 10.1155/2017/4173246

**Published:** 2017-12-24

**Authors:** Daniel Vikum, Ingvild Nordøy, Cecilie Torp Andersen, Børre Fevang, Pål Dag Line, Finn Kristian Kolrud, Pål Aukrust, Trond Buanes

**Affiliations:** ^1^Faculty of Medicine, University of Oslo, Oslo, Norway; ^2^Section of Clinical Immunology and Infectious Diseases, Oslo University Hospital, Oslo, Norway; ^3^Research Institute of Internal Medicine, Oslo University Hospital, Oslo, Norway; ^4^Department of Microbiology, Oslo University Hospital, Oslo, Norway; ^5^Section for Transplantation Surgery, Department of Transplantation Medicine, Oslo University Hospital, Oslo, Norway; ^6^Department of Hepato-Pancreatico-Biliary Surgery, Oslo University Hospital, Oslo, Norway; ^7^Department of Radiology, Oslo University Hospital, Oslo, Norway

## Abstract

A 24-year-old woman with coeliac disease and transient neutropenia developed mucormycosis with extensive involvement of the liver and small intestine. She was successfully treated with aggressive surgical debridements and long-term antifungal therapy with liposomal amphotericin B and posaconazole.

## 1. Introduction

Mucormycosis is an uncommon but emerging angioinvasive infection. It is caused by fungi in the order of Mucorales, predominantly affects immunocompromised individuals, and carries a dismal prognosis. Gastrointestinal infection is a rare presentation, and there are few reports on hepatic mucormycosis. We report the case of a 24-year-old woman with coeliac disease and transient neutropenia during phenoxymethylpenicillin treatment, who developed gastrointestinal mucormycosis with extensive hepatic abscess formation.

## 2. Case Report

A 24-year-old woman with substituted vitamin B12 deficiency and coeliac disease presented with an infected molar tooth and tonsillitis unresponsive to 4-day treatment with phenoxymethylpenicillin. Blood tests at hospital admission (day 1) revealed a neutrophil count of 0.0 × 10^9^/L. Therapy with penicillin and metronidazole was initiated before transferal to our hospital on day 3 with suspicion of leukemia.

On arrival at our hospital, she was afebrile and, apart from a painful tooth, in good clinical condition. Laboratory evaluation showed hemoglobin 11.0 mg/dL, leucocytes 0.5 × 10^9^/L, platelets 309 × 10^9^/L, ferritin 461 *μ*g/L, fibrinogen 7.1 mg/dL, D-dimer 2.0 *μ*g/mL, albumin 25 g/L, and C-reactive protein 387 mg/mL. Creatinine and liver enzymes were within normal limits. Blood cultures taken at day 3 grew *P. aeruginosa*. No evidence of malignancy or hematological disease was found. A bone marrow biopsy showed granulocytic maturation arrest, a documented side effect of antibiotics.

**Figure 1:**
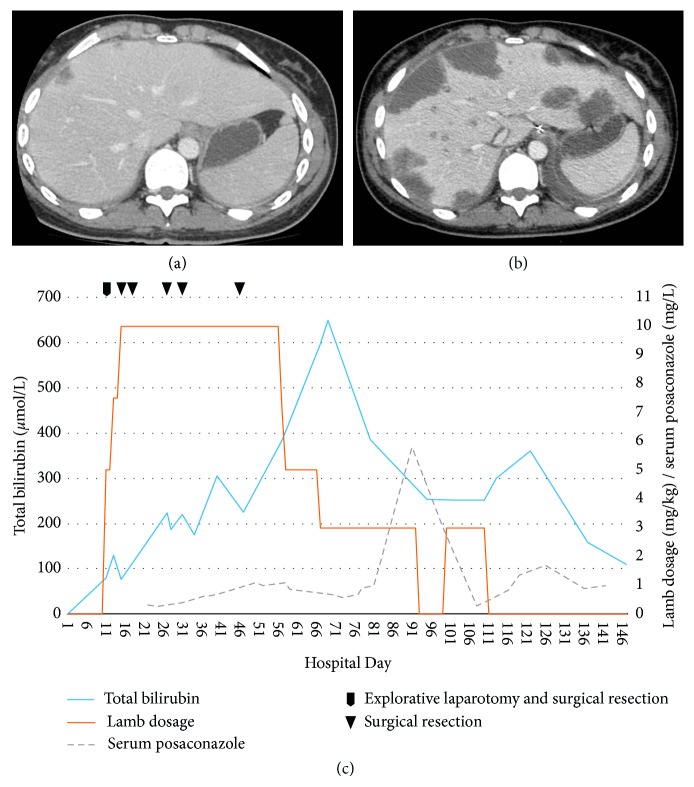
(a) Portal venous phase contrast-enhanced CT shows several small hypoattenuating lesions with slight peripheral enhancement. (b) Two days later, CT shows progression with multiple large hypoattenuating lesions in both the left and right lobe of the liver, suggestive of liver abscesses. (c) Time course of LAmB dosage, serum posaconazole, and serum bilirubin levels. Taking into account the long serum terminal half-life and mean residence time in the liver tissue of LAmB, the graph suggests that LAmB was an important contributor to the cholestasis and hepatotoxicity (as measured by total bilirubin levels) observed in our patient.

Despite adequate antibacterial treatment for *P. aeruginosa* bacteremia, the patient developed abdominal pain, fever, and respiratory distress on day 10 and was intubated on day 11. On day 12, her neutrophil count increased to 0.5 × 10^9^/L and continued to rise, peaking at 23.4 × 10^9^/L on day 19. Abdominal computed tomography (CT) on day 9 revealed generalized lymphadenopathy, edematous thickening of the cecum, and a few small, hepatic hypoattenuated lesions presumed to represent bacterial abscesses (Figure 1). Repeated abdominal CT on day 11 showed extensive progression of hepatic lesions (Figure 1). The same day, explorative laparotomy was performed with resection of two liver lesions in segments 3 and 6. Histopathologic examination of tissue samples revealed angioinvasive fungal infection with hyphae morphologically consistent with *Mucorales* species. Therapy with liposomal amphotericin B (LAmB) 5 mg/kg/day (increased to 10 mg/kg/day on day 15) and posaconazole 800 mg/day oral suspension was initiated. *Rhizomucor miehei* was identified 6 days later in ascites by DNA sequencing of the internal transcribed spacer 2 and the large subunit RNA gene regions (ITS2 and D1-D2 regions).

Once mucormycosis was deemed a likely diagnosis, a radical strategy for resection of the infected tissue was pursued. On day 15, a right hemihepatectomy, seven local resections in segments 2, 3, and 4, and ileococeal resection of all macroscopically pathological intestinal and mesenteric tissues were performed, leaving her with 2 stomas. *Rhizomucor miehei* was confirmed by DNA sequencing of the resected liver, small intestine, and mesenterial tissue. Based on continuous clinical evaluation and repeated abdominal CT scans, she underwent a total of 5 revisions. Between surgeries, the patient was left with open abdomen and negative pressure dressing, and during days 25–58, she was given intraperitoneal LAmB 50 mg/day. In total, approximately 80% of the liver was removed, leaving a remnant consisting of segment 3 and parts of segments 1 and 2. After day 46, no fungus was detected microscopically in liver biopsies or ascites. Extubation was performed on day 20.

After the last resection, the patient's clinical condition improved, but she was suffering from nausea and hepatic dysfunction with rising bilirubin (peaking at 643 U/L on day 67). Albumin and international normalized ratio levels remained stable. There was no sign of biliary obstruction on radiological examination, and a liver biopsy confirmed fibrosis and intrahepatic cholestasis possibly induced by antifungal drugs. LAmB dosage was reduced, and posaconazole was administered intravenously. Subsequently, bilirubin levels gradually decreased (Figure 1). During hospital week 14 (days 92–98), the bilirubin levels stagnated and increased again. A new liver biopsy revealed the addition of degenerated hepatocytes. LAmB was discontinued at week 16 (day 113). During the next weeks, her liver function tests stabilized. At week 18 (day 121), the patient was discharged to her local hospital on posaconazole tablets. She achieved full clinical recovery shortly after discontinuing posaconazole 5 weeks later. Two years later, she had a normal liver on CT scan. Both intestinal stomas have been reversed.

## 3. Discussion

*Mucorales* are molds ubiquitous in nature. *Rhizomucor* spp. have been found contaminating air, soil, water and organic matter worldwide [1] and have also been recovered from tap water in the paediatric stem cell transplantation unit at our hospital [2]. *Rhizomucor miehei* is not commonly associated with human disease, and its specific epidemiology is unknown, but several publications have noted its potential for human infection [1]. A literature review by Gomes et al. in 2011 found 22 case reports of *Rhizomucor* spp. infection with sufficient clinical information to provide definite identification at the species level; in all of these cases, *Rhizomucor pusillus* was the infecting agent [1]. The same review found one case of invasive *Rhizomucor miehei* infection in the literature occurring in a transplant recipient [3]. However, no clinical description is available for this case. The major modes of transmission for human *Mucorales* infection include inhalation, ingestion, and cutaneous exposure [4]. In our patient, the concomitant finding of *Rhizomucor miehei* infection in the liver tissue and small bowel without any sign of pulmonary involvement strongly suggests a gastrointestinal portal of entry.

The most common conditions predisposing to mucormycosis are neutropenia, other forms of immunosuppression, diabetes, and penetrating trauma [5]. Other risk factors include iron overload, prematurity, malnourishment, and illicit intravenous drug use [6]. Apart from transient neutropenia, our patient had no known predisposing risk factors for mucormycosis. Despite an extensive search—including whole genome sequencing—no unknown underlying disease or immunodeficiency was identified. Based on findings in bone marrow biopsy, her neutropenia was probably penicillin induced and as such only limited to days before the *Mucorales* infection occurred. A reduced mucosal immunity due to her coeliac disease may have contributed to the invasion of *Mucorales* during transient neutropenia. To the best of our knowledge, there is no literature documenting an association between coeliac disease and *Mucorales* infection, nor any data on the frequency of coeliac disease amongst patients who have developed this infection. Her initial molar infection, which evolved to sepsis, may be yet a contributing factor enhancing *Mucorales* entry from the gut.

The most common presentations of *Mucorales* infection are rhino-orbital-cerebral, pulmonary, cutaneous, gastrointestinal, or disseminated disease [7]. In a review of 929 reported cases of mucormycosis between 1940 and 2003, gastrointestinal infection represented 7% of the cases and was associated with 85% mortality [7]. In 230 patients with mucormycosis included in a recent prospective study by Skiada et al., only one patient had hepatic infection. Combined small bowel and hepatic mucormycosis has previously been reported in the context of febrile neutropenia after chemotherapy. Few cases of hepatic infection in the immunocompetent host have been published [8, 9].

The diagnosis of gastrointestinal mucormycosis is usually delayed or obtained postmortem due to a nonspecific presentation requiring a high index of suspicion and early biopsy [6]. Our patient had typical CT findings of hepatic mucormycosis with several hypodense hepatic lesions surrounding vessels with no mass effect representing the necrotic tissue due to angioinvasion and fungal thrombosis [10]. The finding was initially interpreted as bacterial abscesses, but rapid progression on repeat CT scan after only 2 days was an important diagnostic clue and a characteristic feature of invasive mucormycosis.

Histopathologic examination of tissues with *Mucorales* infection typically shows characteristic broad, ribbon-like hyphae with a few or no septa and wide-angled branching, accompanied by tissue necrosis and angioinvasion [4]. However, direct microscopy with calcofluor-white stain is quicker and gives the same morphological information. Negative tissue cultures are not uncommon, and blood cultures are rarely positive [4]. In our case, the initial diagnosis was based on histopathologic findings and direct microscopy of tissue samples. The diagnosis was confirmed, and *Rhizomucor miehei* was identified with DNA sequencing of tissue samples and ascites. DNA sequencing was not performed on blood. Repeated blood cultures and cultures of tissue samples remained negative for *Mucorales*. The development of new molecular techniques has improved time to diagnosis. After introducing a *Mucorales*-specific real-time polymerase chain reaction assay at our hospital in 2015, *Mucorales* species DNA can be identified the same day tissue samples arrive at the laboratory.

The mainstay of treatment of mucormycosis is (1) antifungal therapy with LAmB, (2) surgical debridement, and (3) reversal of underlying disease. In the study by Skiada et al., factors associated with survival on multivariate analysis were trauma as the underlying condition, treatment with LAmB, and surgery [5]. Aggressive surgical debridement is of major importance because thrombosis and tissue necrosis prevent the penetration of antimycotic agents to the site of infection [4]. In our patient, the amount of liver resected was probably lifesaving. The use of percutaneous catheters alone to drain the *Mucorales* abscesses usually fails to bring resolution [11].

Amphotericin B is the first-line drug for the treatment of mucormycosis [12]. Mortality rates significantly decreased following its introduction in the 1960s but have since remained relatively stable [7]. Newer lipid formulations of amphotericin B are preferable to nonlipid formulations due to significantly reduced nephrotoxicity, allowing the use of higher doses. A phase I-II study reported that dose escalation of LAmB beyond 10 mg/kg/day failed to result in enhanced serum levels, but the authors postulated that infection in tissues like the liver might still benefit from dosages exceeding 10 mg/kg/day, likely due to enhanced uptake by the reticuloendothelial system [13]. LAmB has a long mean residence time in tissues. In one murine study, tissue concentrations of LAmB in the liver increased over 2 weeks after treatment [14].

Posaconazole, a triazole, is recommended as the second-line or salvage therapy in cases refractory to amphotericin B [12]. Delayed-release tablets and intravenous formulations of posaconazole are now available ensuring better bioavailability. Isavuconazole is the only azole approved by the Food and Drug Administration for the treatment of invasive mucormycosis, but clinical data about its use in mucormycosis are scarce [15]. Proper studies on the use of combination therapy are lacking.

In conclusion, in a patient with coeliac disease, molar infection, and a probable penicillin-induced transient neutropenia who experienced extensive *Rhizomucor* spp. infection of the liver and intestines, the combination of rapid diagnosis, aggressive and repetitive surgery, and antifungal treatment proved successful.
